# An RFID Indoor Positioning Algorithm Based on Support Vector Regression

**DOI:** 10.3390/s18051504

**Published:** 2018-05-10

**Authors:** He Xu, Manxing Wu, Peng Li, Feng Zhu, Ruchuan Wang

**Affiliations:** 1 School of Computer Science, Nanjing University of Posts and Telecommunications, Nanjing 210023, China; 1217043221@njupt.edu.cn (M.W.); lipeng@njupt.edu.cn (P.L.); zhufeng@njupt.edu.cn (F.Z.); wangrc@njupt.edu.cn (R.W.); 2 Jiangsu High Technology Research Key Laboratory for Wireless Sensor Networks, Nanjing 210003, China

**Keywords:** indoor positioning, LANDMARC, RFID

## Abstract

Nowadays, location-based services, which include services to identify the location of a person or an object, have many uses in social life. Though traditional GPS positioning can provide high quality positioning services in outdoor environments, due to the shielding of buildings and the interference of indoor environments, researchers and enterprises have paid more attention to how to perform high precision indoor positioning. There are many indoor positioning technologies, such as WiFi, Bluetooth, UWB and RFID. RFID positioning technology is favored by researchers because of its lower cost and higher accuracy. One of the methods that is applied to indoor positioning is the LANDMARC algorithm, which uses RFID tags and readers to implement an Indoor Positioning System (IPS). However, the accuracy of the LANDMARC positioning algorithm relies on the density of reference tags and the performance of RFID readers. In this paper, we introduce the weighted path length and support vector regression algorithm to improve the positioning precision of LANDMARC. The results show that the proposed algorithm is effective.

## 1. Introduction

In recent years, the rise of the Internet of Things (IoT) has brought great convenience to our lives [1]. By means of a local network or Internet communication technology, we can connect sensors, controllers, machines, persons and objects through IoT technology to obtain information, for remote management, control and services. At present, object location awareness is a very important part of the IoT’s application, but it cannot obtain this through the traditional satellite and cellular location with GPS in indoor environments [2,3]. In order to implement indoor positioning, there are many different technologies, such as WiFi [4], UWB [5], Radio Frequency Identification (RFID) [6], and so on.

RFID is regarded as the key technology to implement IoT [7], and RFID technology can be used in many fields, such as warehouse asset management, product tracking, supply chain management, anti-counterfeiting identification, healthcare, and so on. With the development of RFID technology, RFID indoor positioning has been selected as one of the indoor wireless location technologies, and its development has been rapid [8]. RFID technology has many advantages for positioning, such as low cost, lightweight and multiple tag identification. However, there are still some problems to be solved for RFID indoor positioning [9]. In the following, we mainly review some RFID-based indoor positioning technologies.

The RFID technology positioning system usually consists of antennas, tags, readers and positioning algorithms. The following algorithms are among the RFID-based indoor positioning algorithms: Time of Arrival (TOA), Time Difference of Arrival [10] (TDOA), Angle of Arrival (AOA) and Received Signal Strength Indication (RSSI). Compared to TOA and TDOA, the RSSI-based RFID positioning algorithm does not require a strict synchronization mechanism between the tag and the reader, making it easy to implement, and its positioning accuracy is within a reasonably acceptable range. The AOA positioning technology uses the angular position of the received signal to locate the position of the label based on the angle of arrival of the multiple signals and the triangulation algorithm. However, positioning by the AOA technique is greatly influenced by the non-line-of-sight effect; therefore, it cannot be applied to complex indoor environments. In addition, readers equipped with antenna arrays during AOA positioning are not only resource-intensive, but also costly. Table 1 gives a comparison of the above indoor positioning algorithms with the fingerprint and reference tag methods.

Because passive UHF RFID has a low deployment cost and its rationality has been verified, it is widely used in many areas. Buffi et al. introduced and validated a new phase-based technology for locating and tracking items moving along a conveyor belt [11], which were equipped with UHF-RFID tags, and the positioning performance and accuracy were verified in the real conveyor case. The results showed that centimeter-accurate positioning of tags can be achieved even in very complex multi-path environments. To overcome the limitations of current RFID systems, Guidi et al. suggested that RFID and ultra-wideband (UWB) tag technology [12,13] could be used in combination to reduce the positioning error due to the large bandwidth involved. In the above two references, the authors conducted in-depth research on the application of backscatter signals in positioning. Arnitz et al. provided a proof for a UWB-based passive UHF RFID [14] ranging concept, which was very robust to multipath propagation. Guidi et al. also proposed a specific assignment strategy [15] to simplify the acquisition of the tag code at the reader side, which verified the feasibility of the excellent real-time multi-tag positioning. Li et al. analyzed the different multi-path interferences of a needle under the storage scenario and analyzed the frequency bands suitable for different environments and accuracy requirements [16].

One of the most famous RFID positioning algorithms is LANDMARC [17]. The LANDMARC system is a typical reference-tag-based RFID positioning method proposed by a research group of the Hong Kong University of Science and Technology. By introducing a reference label to assist positioning, a reference label matrix is arranged in the positioning space; RSSI values of the unknown label and the reference label are measured; and the nearest neighbor K value algorithm is used for positioning. However, the number of reference tags is huge. The introduction of a large number of these reference labels will also require many unnecessary calculations, as well as reduce the positioning accuracy.

Many scholars have proposed many methods to improve the positioning accuracy of the LANDMARC indoor positioning algorithm. He Xu et al. proposed an algorithm that was based on the Bayes and kNN [18] algorithm to improve the indoor location accuracy [19]. The result of the kNN algorithm was greatly influenced by the k value [20]. When the k value was too small, the model was too complicated, resulting in an over-fitting phenomenon. On the contrary, when the k value was too large, a great deal of useful information in the training examples would be ignored. Yongjun Zhang proposed the BPNN-LANDMARC method in [21], which was an improved indoor positioning algorithm based on the BP neural network. The algorithm reduced the time complexity of prediction, but the neural network training and tuning were very complicated processes, which required a large workload. In addition, there have been other improvements based on deep learning [22,23] to improve position accuracy. In this paper, we will use the Support Vector Regression (SVR) method and weighted path length to improve the accuracy of traditional LANDMARC positioning. The advantage is that the Gaussian-Kalman filter is used to solve the noise, and SVR is used to predict positions and thus improve the accuracy of positioning compared to the traditional RFID positioning methods.

This paper is structured as follows: Section 1 introduces related research results about indoor position technology and RFID-based indoor positioning algorithms. Section 2 details the background knowledge of LANDMARC. Section 3 gives the Gaussian-Kalman filter theorem used in the proposed algorithm. In Section 4, an indoor positioning algorithm based on SVR is proposed where the Gaussian-Kalman filter is used to deal with the noise in RSSI signals. Section 5 evaluates the performance of the Gaussian-Kalman filter and the presented algorithm with other indoor positioning algorithms in the experiments. Finally, we conclude the paper.

## 2. Background Knowledge

### 2.1. Logarithmic Distance Loss Model

In the wireless channel, the distance between the RSSI transmitting antenna (reader’s side) and the receiving antenna (tag’s side) is generally expressed by the following formula:

(1) $$PL\left(d\right)=PL\left(d_{0}\right)+10n\log\left(\frac{d}{d_{0}}\right)+X_{\sigma }\left(dB\right)$$

where $d_{0}$ is the reference distance, which is usually set as one meter. PL(d) is the path loss of the wireless signal in free space when the propagation distance is $d_{0}$. $X_{\sigma }$ is usually called shadow fading. σ is a Gaussian random variable. n is the path loss index. Equation (1) is usually used to estimate the path loss of the propagation of wireless signals in indoor environments due to obstacles, moving objects and the antenna directivity between the tag and reader. If we assume that the distance between the transmitting antenna and the receiving antenna is d and the signal strength value received by the terminal is set to $P_{r}\left(d\right)$, and $\omega =P_{r}-PL\left(d_{0}\right)$, then,

(2) $$P_{r}\left(d\right)=P_{r}-PL\left(d_{0}\right)=\omega -10n\log\left(\frac{d}{d_{0}}\right)+X_{\sigma }\left(dB\right)$$

In the indoor positioning algorithm, Equation (2) is a commonly-used wireless channel modeling method, where n is the path loss index in different environments and σ is the variance.

### 2.2. The Principle of the LANDMARC Algorithm

The LANDMARC algorithm is a typical scene analysis method, which also is a proactive calibration algorithm. By introducing the concept of the reference label, it is used as a reference point to provide positioning constraints. The main advantage of this approach is the replacement of a large number of expensive RFID readers with additional and less expensive reference tags. The indoor positioning system using the LANDMARC algorithm helps to locate things by adding a large number of reference labels. The topological structure of reference labels and the number of nearest labels in the algorithm are both important factors in determining the positioning accuracy. The main idea of the LANDMARC algorithm is to find several reference labels with the closest Euclidean distance to unknown labels, by recording the RSSI values of the labels to be tested and the reference labels and calculating the Euclidean distance of the electromagnetic features involved.

The following describes the LANDMARC algorithm in mathematical notation.

The method used to find the Euclidean distance to an unknown label in the LANDMARC algorithm is called the kNN algorithm. It is assumed that the positioning situation contains one reader, m reference labels and n labels to be tested, where the reader operates in continuous mode. The location of the tag to be located is calculated based on the actual location of the neighbor reference tags and the weight values. The signal strength vector of the target label is defined as $S=\left(s_{1},s_{2},s_{3},\ldots ,s_{n}\right)$, and the signal strength vector of the reference label is $\theta =\left(\theta_{1},\theta_{2},\theta_{3},\ldots ,\theta_{n}\right)$. The Euclidean distance of the signal strength between the target label and the reference label is defined as follows:

(3) $$E_{j}=\sqrt{\overset{n}{\underset{i=1}{\sum }}\left(\theta_{i}-s_{i}\right)}\;j\in \left(1,m\right)$$

In Equation (3), E represents the distance between the reference label and the target label. The smaller the value of E is, the closer the distance between the reference label and the target label is. For m reference labels, the signal strength level vector $E=\left(E_{1},E_{2},E_{3},\ldots ,E_{m}\right)$, LANDMARC will select k reference labels with the smallest E value and finally locate the target label by the weight-centroid algorithm. In this algorithm, if the distance between the reference label and the target label is closer, the signal strength read by the reader will be closer to the value, and the larger E is, the closer the distance between the reference label and the target label is.

### 2.3. Weighted Path Length

The weighted path length compares the RSSI value of the reference tags and the target label received by each reader and determines k neighboring reference tags according to the signal strength value of the tag with the positioning tag. These *k* reference labels can determine a polygon, and the centroid of this polygon is taken as the position of the target tag; which is as follows:

(4) $$\left(x,y\right)=\overset{k}{\underset{i=1}{\sum }}w_{i}\left(x_{i},y_{i}\right),w_{i}=\frac{\frac{1}{E_{i}^{2}}}{\sum_{i=1}^{k}\frac{1}{E_{i}^{2}}}$$

The coordinates of the two dimensions of the unknown labels are calculated separately by the Equation (4), which is the indoor positioning process of the traditional LANDMARC algorithm.

## 3. Gaussian-Kalman Filter

The RFID system can read much electronic tag information in a short time. However, the reading RSSI values can be affected by various factors such as indoor temperature, humidity and multipath effects, so data preprocessing is required.

### 3.1. Gaussian Filter

For the same tag, due to various aspects of interference, the RSSI value received by the reader has a small probability of error data and a large probability of accurate data. The Gaussian mathematical model is used to select the RSSI value of the large probability range as the effective value of the data sampling and then calculate the average value as the output of the filtering. This method can effectively reduce the impact of low probability and strong interference on the overall data and improve the data accuracy.

The RSSI measurement follows the Gaussian distribution of $\left(0,\sigma^{2}\right)$, and its probability density function is shown in Equation (5).

(5) $$f=f_{0}+\frac{1}{\omega \sqrt{\frac{\pi }{2}}}.e^{-2\frac{{\left(x-x_{e}\right)}^{2}}{\omega^{2}}}$$

In Equation (5), $x_{e}=\frac{1}{k}.\sum_{i=1}^{k}RSSI_{i},\;\omega =\sqrt{\frac{1}{k-1}.\sum_{i=1}^{k}{\left(RSSI_{i}-x_{e}\right)}^{2}}$.

Firstly, Gaussian filtering is used to put the RSSI measurement data into the fitting function, so as to obtain large probability data and then calculate the average of the retained RSSI values, and the obtained value is the determined RSSI value. However, the calculation volume is too large, and the anti-interference ability is poor. Therefore, the approximate Gaussian fitting method is used to pre-process the data. The baseline and standard deviation are the average of the original measurement data, which are calculated as in Equation (6), given as:

(6) $$\left|RSSI_{i}-x_{e}\right|<k\omega$$

where k can be determined according to the percentage (*P*) of the data that needs to be retained. The value of *P* is calculated using the following Equation (7):

(7) $$P=\frac{K}{N}\times 100\%$$

where *K* is the number of reserved data and *N* is the total of the sampled data. Obviously, if the P value is small, it means that there are few samples of the reserved data, which will reduce the authenticity of the data. Conversely, if the P value is too large, the wrong data may remain. According to the fluctuation of RSSI, the value of *K* should be changed appropriately. This will not only increase the speed of calculation, but also effectively eliminate the mutation of data.

Gaussian fitting can only filter data and does not eliminate data fluctuations. In order to eliminate the fluctuation of RSSI data and make them a smooth output, the Kalman filter algorithm can be used. Gaussian filtering can solve the problems of the RSSI value being vulnerable to interference and bad stability. It can improve the positioning accuracy to a certain extent, but its effect on long-term error interference such as energy reflection and the multipath effect is not good.

### 3.2. Kalman Filter

The basic idea of Kalman filtering: Take the minimum mean square error as the best estimation principle, using the signal and noise state model, using the observations at the current moment and the estimates at the previous moment to update the estimation of the state variables, so as to calculate the current estimated value. Based on the established observation equations and system of equations, the RSSI value is estimated as the minimum mean square error.

Assume that the RSSI is represented by $x_{k}$, and the sampled data model can be represented by a first-order self-recursive equation of a Gaussian white noise sequence. The equation of state and observation equation are represented by Equations (8) and (9).

Equation of state:

(8) $$x_{k}=\varphi_{k,k+1}.x_{k-1}+\omega_{k-1}$$

Observation equation:

(9) $$y_{k}=x_{k}+v_{k}$$

where $x_{k}$ is the RSSI value at time *k*; $y_{k}$ is the observed value of $x_{k}$ at time *k*; $\omega_{k-1}$ and $v_{k}\left(k\in N\right)$ are independent and identically distributed noise sequences; the Gaussian white noise sequence with the dominant change of $\omega_{k-1}$; $v_{k}$ is the noise during measurement at time *k*; where $\omega_{k-1}\sim N\left(0,Q\right)$, $v_{k}\sim N\left(0,R\right)$. Q represents the process excitation noise covariance matrix, and R represents the observation noise covariance matrix.

The time update equation is shown in Equation (10):

(10) $$\{\begin{array}{l} P_{k,k-1}=\varphi_{k,k-1}.P_{k-1}.\varphi_{K,K-1}^{T}+R_{\omega K-1}\\ {\hat{x}}_{k+1,k}=\varphi_{k+1,k}.{\hat{x}}_{k} \end{array}$$

The measurement update equation is shown in Equation (11):

(11) $$\{\begin{array}{l} {\hat{x}}_{k}=\varphi_{k,k-1}.{\hat{x}}_{k-1}+K_{k}.\left(y_{k}-\varphi_{k,k-1}.{\hat{x}}_{k-1}\right)\\ K_{k}=P_{k,k-1}.{\left(P_{k,k-1}+R_{vk}\right)}^{-1}\\ P_{k}=\left(I-K_{k}\right).P_{k,k-1} \end{array}$$

where $P_{k,k-1}$ denotes the prediction mean squared error matrix; ${\hat{x}}_{k+1}$ denotes the state of the next step predictor; ${\hat{x}}_{k}$ denotes the state estimate; $K_{k}$ denotes the filter gain matrix; $P_{k}$ denotes the filter mean square error matrix.

The advantage of the Kalman filter is that it can reduce the error of RSSI caused by noise superposition. The stability of filtered RSSI is good, and the Kalman filter is especially suitable for data processing in target tracking and positioning. The main advantage of the Kalman filtering algorithm is that it can obtain smooth data output. The implementation of the process prediction and correction phase is as follows.

The prediction stage is as Equations (12):

(12) $$\{\begin{array}{l} {\hat{x}}_{k+1,k}=\varphi_{k+1,k}.{\hat{x}}_{k,k}\\ Q_{x\left(k+1,k\right)}=\emptyset_{k+1,k}Q_{x\left(k+1,k\right)}\emptyset_{k+1}^{T}+\Gamma_{k+1,k}^{T}Q_{x\left(k,k\right)}\Gamma_{k+1,k}^{T} \end{array}$$

The correction stage is as Equations (13):

(13) $$\{\begin{array}{l} {\hat{x}}_{k+1,k+1}={\hat{x}}_{k+1,k}+\left({\hat{L}}_{k+1}-B_{k+1}{\hat{x}}_{k+1,k}\right)\\ Q_{x\left(k+1,k+1\right)}=\left(1-K_{k+1}B_{k+1}\right)Q_{x\left(k+1,k\right)}\\ K_{k+1}=Q_{x\left(k+1,k\right)}B_{k+1}^{T}+\left[B_{k+1}Q_{x\left(k+1,k\right)}B_{k+1}^{T}+Q_{L\left(k+1\right)}\right] \end{array}$$

**Figure 1:**
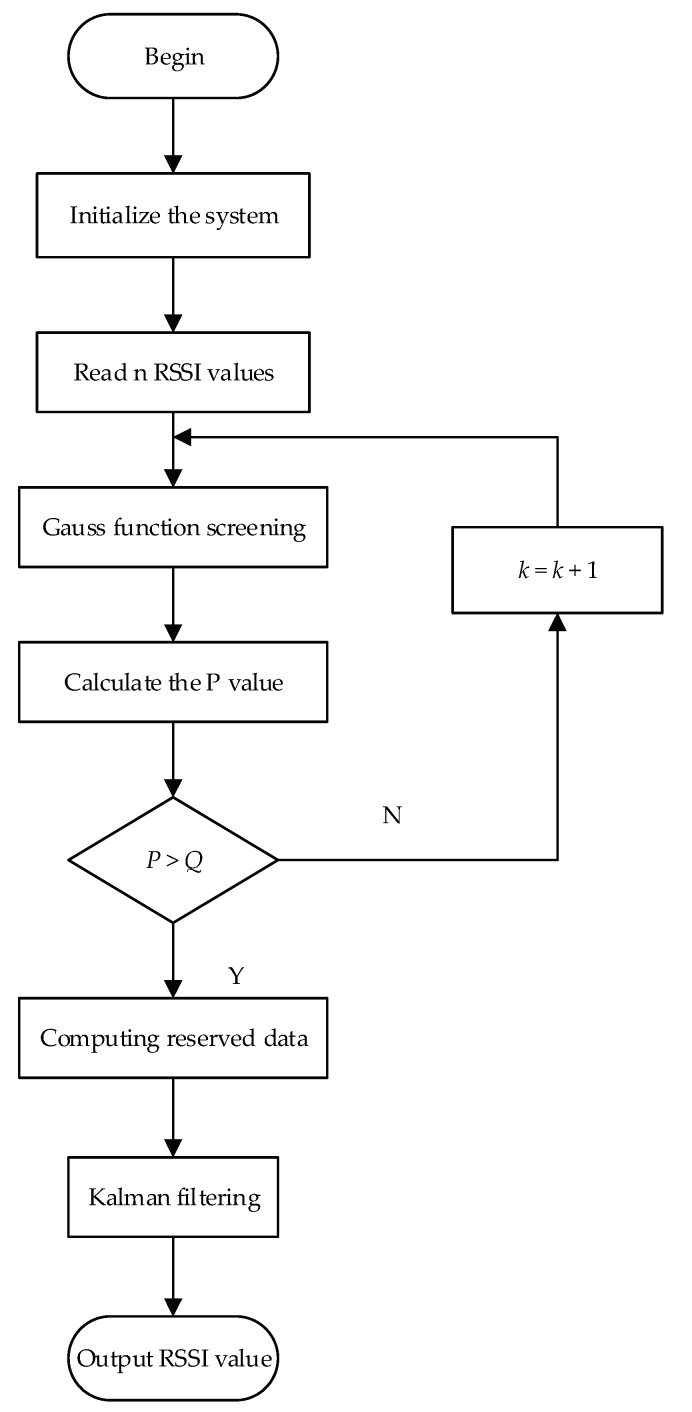
Flowchart of the Gaussian-Kalman filter algorithm.

In the above formula, ${\hat{x}}_{k,k}$ represents the state estimate of RSSI at time *k*; ${\hat{x}}_{k+1,k}$ represents the next predicted value of RSSI at time K; ${\hat{L}}_{k+1}$ represents the prediction of the *k* + 1 time; $B_{k+1}$ denotes the predictive matrix; $Q_{x\left(k+1,k\right)}$ denotes the variance matrix of the prediction error estimate for the k+1 moment at *k* time. $Q_{x\left(k+1,k+1\right)}$ represents a variance matrix; $K_{k+1}$ represents a gain matrix. The Gaussian-Kalman filter algorithm flowchart is shown in Figure 1.

The details of the Gaussian-Kalman filter implementation process are listed as follows:

(1) Initialize parameters n, *Q*, *K*, *P*. n represents the total RSSI data sample; *Q* represents the lowest percentage of screening data; K is the screening interval coefficient; and *P* is the percentage of data retention.
(2) If *P* < *Q*, the *K* value increases, and the RSSI value is put into Gaussian fitting once again. If *P* > *Q*, the standard deviation σ and the mean $x_{e}$ of the Gaussian parameters are preserved.
(3) Kalman filtering is used to perform the Gaussian fit-filtering of the RSSI value, and the average value of filtered RSSI is selected as the input of the positioning algorithm to calculate the positioning result.

## 4. The Improved SVR-LANDMARC Algorithm

Support vector regression is a regression method that is similar to least squares [24], ridge regression [25] and gradient descent. Support Vector Machines [26,27] (SVM) also comprise another method that can support vector regression. Support Vector Regression (SVR) uses the support vector and the Lagrange multiplier approach for the regression analysis for data. It has many advantages over the least squares method, which is shown in the following.

1. The least squares method can only be used for linear regression, but not for nonlinear models. However, support vector regression does not have this limitation.
2. The least squares method does not work well for regression with variables that have multiple collinearities, while support vector regression can process multicollinearity well.
3. Although support vector regression does not directly exclude the anomaly in the process, it can make the error caused by the anomalous point smaller.

Regression and classification are, in a sense, essentially the same method. The idea of SVM classification is to find a plane that allows the support vectors of all two classification sets or all data to be furthest away from the classification plane. The idea of SVR regression is to find a regression plane so that all data of a set are closest to it. The following details the SVR principle.

- Principle of SVR

Given training samples $D=\left\{\left(x_{1},y_{1}\right),\left(x_{2},y_{2}\right),\ldots ,\left(x_{m},y_{m}\right)\right\}$, $y_{i}\in \mathbb{R}$; where $x_{i}$ is the vector of RSSI values read from the same reader to the same tag and $y_{i}$ is a value in a two-dimensional coordinate of a reference label (the two values of two-dimensional coordinates are separately fitted). SVR will learn a model using the following Equation (14).

(14) $$f\left(x\right)=\omega^{T}x+b$$

**Figure 2:**
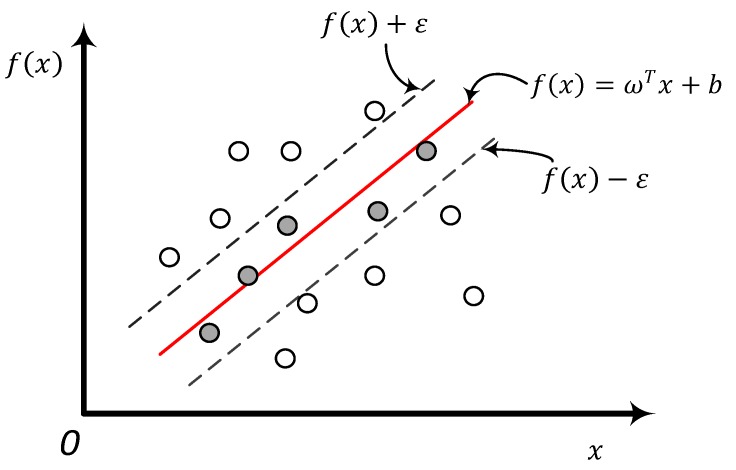
Support vector regression diagram.

The goal is to make f(x) as close to the true value y as possible. In Equation (14), ω and b are the model parameters to be determined. For the sample (x,y), the traditional regression model usually calculates the loss directly based on the difference between the model output f(x) and the true value y. The loss is zero only if the predicted value f(x) is exactly the same as the true value y. In contrast, it can be assumed that support vector regression can tolerate at most ε deviations between predicted value f(x) and true value y. That is to say, the loss is calculated only if the absolute value of the difference between f(x) and y is greater than ε. As shown in Figure 2 below, this is equivalent to constructing an interval with a width of 2ε centered on f(x). If a training sample falls into this interval, it is considered as being correctly predicted.

Thus, the SVR problem can be transformed into the following Equation (15):

(15) $$\underset{\omega ,b}{\min}\;\frac{1}{2}{\left|\left|\omega \right|\right|}^{2}+C\overset{m}{\underset{i=1}{\sum }}l_{\varepsilon }\left(f\left(x_{i}-y_{i}\right)\right)$$

where C is a regularized constant, ε indicates that the SVR algorithm can tolerate the most ε deviation between the predicted value *f*(*x*) and the real value *Y*. That is, only when the absolute value of the difference between the *f*(*x*) and the *Y* is greater than the ε, the loss is calculated. ω is the normal vector of the SVR algorithm to fit the hyperplane. $l_{\epsilon }$ is the ε-insensitive loss function shown in Figure 2.

(16) $$l_{\epsilon }=\{\begin{array}{c} 0,\;\;if\;\left|z\right|\leq \varepsilon ;\\ \left|z\right|-\varepsilon ,\;\;otherwise \end{array}$$

In fact, Equation (16) shows the function distance between the regression function and the coordinate value of one dimension of the reference label of the actual training point. For the loss function defined in the formula, the meaning of the expression is to allow the model to have a certain error. All points within the error range are considered as points on the model. Additionally, only those points on the boundary beyond the error range are the support vectors that determine the model. Because of the similar flexible boundary in SVM, we introduce slack variable $\xi_{i}$ and $\hat{\xi_{i}}$ and consider the error function (15) as a convex optimization problem.

(17) $$R\left(\omega ,\xi_{i,},\hat{\xi_{i}}\right)=\underset{\omega ,b,\xi_{i,},\hat{\xi_{i}}}{\min}\;\frac{1}{2}{\left|\left|\omega \right|\right|}^{2}+C\overset{m}{\underset{i=1}{\sum }}\left(\xi_{i}+\hat{\xi_{i}}\right)$$

It is subject to the following Equation (18):

(18) $$f\left(x_{i}\right)-y_{i}\leq \epsilon +\xi_{i},\;y_{i}-f\left(x_{i}\right)\leq \epsilon +\hat{\xi_{i}},\;\xi_{i}\geq 0,\hat{\xi_{i}}\geq 0,\;i=1,2,\ldots ,\;m.$$

The larger the C is, the greater the penalty of the data samples is, the difference of which is between the predicted value and the actual value. Therefore, the choice of parameter C is especially important. Its value affects the generalization ability of the system. The smaller the value of ε, the higher the accuracy of the function regression, and the greater the number of support vectors. The greater the value of ε, the fewer the number of support vectors and the lower the regression accuracy. Therefore, according to the nature of the sample, choosing reasonable values of C and ε is helpful to increase the accuracy of model prediction. In Equation (17), both $\xi_{i}$ and $\hat{\xi_{i}}$ are larger than zero when the division is wrong. When the error does not exist, $\xi_{i}$ and $\hat{\xi_{i}}$ take zero. At this point, the problem translates into the problem of minimizing the objective function (17). The first term in (17) makes the fitting function flatter, which can improve the generalization ability to predict the coordinates of unknown labels. The second is to reduce the error. The constant C > 0 indicates the degree of penalty for samples that exceed the error ε. Lagrange function is introduced to solve the optimization problems of (17) and (18):

(19) $$L=\frac{1}{2}{\left|\left|\omega \right|\right|}^{2}+C\overset{m}{\underset{i=1}{\sum }}\left(\xi_{i}+\hat{\xi_{i}}\right)-\overset{m}{\underset{i=1}{\sum }}\alpha_{i}\left[\xi_{i}+\varepsilon -y_{i}+f\left(x_{i}\right)\right]-\overset{m}{\underset{i=1}{\sum }}\hat{\alpha_{i}}\left[\hat{\xi_{i}}+\varepsilon -y_{i}+f\left(x_{i}\right)\right]-\overset{m}{\underset{i=1}{\sum }}\left(\xi_{i}\gamma_{i}+\hat{\xi_{i}}\hat{\gamma_{i}}\right)$$

where $\alpha_{i},\hat{\alpha_{i}}\geq 0$, $\gamma_{i},\hat{\gamma_{i}}\geq 0$, L is the Lagrange function and i=1,2,…,m. Find the function L to minimize $\omega ,b,\xi_{i},\hat{\xi_{i}}$ and maximize $\alpha_{i},\hat{\alpha_{i}},\gamma_{i},\hat{\gamma_{i}}$, and bring them into the Lagrange function to get the dual form, so as to maximize the value of the function.

(20) $$W\left(\alpha_{i},\hat{\alpha_{i}}\right)=\frac{1}{2}\overset{m}{\underset{i=1,j=1}{\sum }}\left(\alpha_{i}-\hat{\alpha_{i}}\right)\left(\alpha_{j}-\hat{\alpha_{j}}\right)\left(x_{i}*x_{j}\right)+\overset{m}{\underset{i=1}{\sum }}\left(\alpha_{i}-\hat{\alpha_{i}}\right)y_{j}-\overset{m}{\underset{j=1}{\sum }}\left(\alpha_{j}-\hat{\alpha_{j}}\right)\varepsilon$$

subject to,

(21) $$\{\begin{array}{l} \overset{m}{\underset{i=1}{\sum }}\left(\alpha_{i}-\hat{\alpha_{i}}\right)=0\\ 0\leq \alpha_{i},\hat{\alpha_{i}}\leq C \end{array}$$

Solving (20), (21) is also a problem for solving quadratic programming. According to the Kuhn–Tucker theorem, the following conclusions are drawn at the saddle point.

(22) $$\hat{\alpha_{i}}\left[\hat{\xi_{i}}+\varepsilon -y_{i}+f\left(x_{i}\right)\right]=0\;\alpha_{i}\left[\xi_{i}+\varepsilon -y_{i}+f\left(x_{i}\right)\right]=0\;\xi_{i}\gamma_{i}=0\;\hat{\xi_{i}}\hat{\gamma_{i}}=0$$

It is obtained $\alpha_{i}*\hat{\alpha_{i}}=0$, that is to say, $\alpha_{i},\hat{\alpha_{i}}$ cannot be zero at the same time, which can also be drawn as below.

(23) $$\left(C-\alpha_{i}\right)\xi_{i}=0\;\left(C-\hat{\alpha_{i}}\right)\hat{\xi_{i}}=0$$

From Equation (23), it can be concluded that $\left|f\left(x_{i}\right)-y_{i}\right|$ may be larger than ε when $\alpha_{i}=C$ or $\hat{\alpha_{i}}=C$, and its corresponding $x_{i}$ is called the boundary support vector. It corresponds to the dotted line in Figure 2. When $\hat{\alpha_{i}}\in \left(0,C\right)$, $\left|f\left(x_{i}\right)-y_{i}\right|=\varepsilon$, that is, $\xi_{i}=0$, $\hat{\xi_{i}}=0$, the corresponding $x_{i}$ is called the support vector. It corresponds to the data points in Figure 2 falling on the ε band. When $\alpha_{i}=0$, $\hat{\alpha_{i}}=0$, the corresponding $x_{i}$ is a non-support vector, corresponding to the points in the ε-band in Figure 2, which do not contribute to ω. Therefore, the larger ε, the smaller the number of support vectors. For the standard support vector, b can be obtained from Equation (24) if $0<\alpha_{i}<C\left(\hat{\alpha_{i}}=0\right)$ and $\xi_{i}=0$.

(24) $$b=y_{i}-\underset{x_{j}\in SV}{\sum }\left(\alpha_{i}-\hat{\alpha_{i}}\right)x_{i}*x_{j}-\varepsilon$$

The values of b are generally calculated for all standard support vectors, and then averaged, that is:

(25) $$b=\frac{1}{N_{NSV}}\left\{\underset{0<\alpha_{i}<C}{\sum }\left[y_{i}-\underset{x_{j}\in SV}{\sum }\left(\alpha_{i}-\hat{\alpha_{i}}\right)K(x_{i},x_{j}\right)-\varepsilon ]+\underset{0<\hat{\alpha_{i}}<C}{\sum }\left[y_{i}-\underset{x_{j}\in SV}{\sum }\left(\alpha_{i}-\hat{\alpha_{i}}\right)K(x_{i},x_{j}\right)-\varepsilon ]\right\}$$

Therefore, sample point $\left(x_{i},y_{i}\right)$ can be used to find the linear fit function as the following formula:

(26) $$f\left(x_{i}\right)=\omega \times x+b=\overset{m}{\underset{i=1}{\sum }}\left(\alpha_{i}-\hat{\alpha_{i}}\right)x_{i}.x+b$$

Then, the more reasonable model in the positioning environment is obtained by training the reference label. The trained model can predict unknown labels.

## 5. Experiment Results and Performance Evaluation

In this paper, we conducted a series of experiments to verify the improvement of the positioning accuracy by the SVR algorithm. The original LANDMARC algorithm, SA-SVR-LANDMARC [28] and the BPNN-LANDMARC algorithm were compared respectively for 100 trials.

### 5.1. Experimental Environment

The reader used in this paper is Impinj, R420, which supports ISO 18000-6C, output power: 10–32.5 dBm adjustable power. The sensitivity of the receiving antenna is adjustable. The minimum value can reach −82 dBm. The tag is an UHF tag that supports the UHF Gen2 protocol with the feature of the full angle reading of the signal. The size of the tag is 25 mm × 25 mm × 0.1 mm. They are usually used in cargo tracking, access control management, warehousing management, etc. The maximum input power of each radio frequency antenna is 34 dBm; the maximum insertion loss is 1.3 dB; and the minimum return loss is 24 dB. According to [29], the RFID antenna model used in our experiments is that the antenna of the RFID reader has a circular shape.

**Figure 3:**
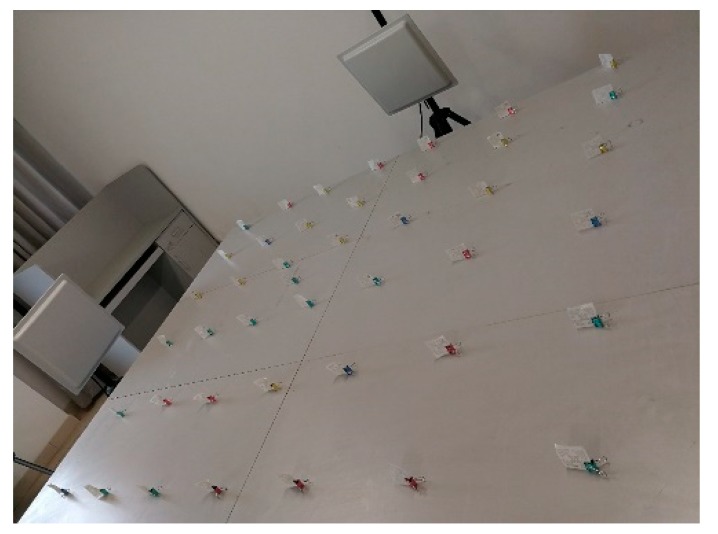
Part of the experimental environment.

**Figure 4:**
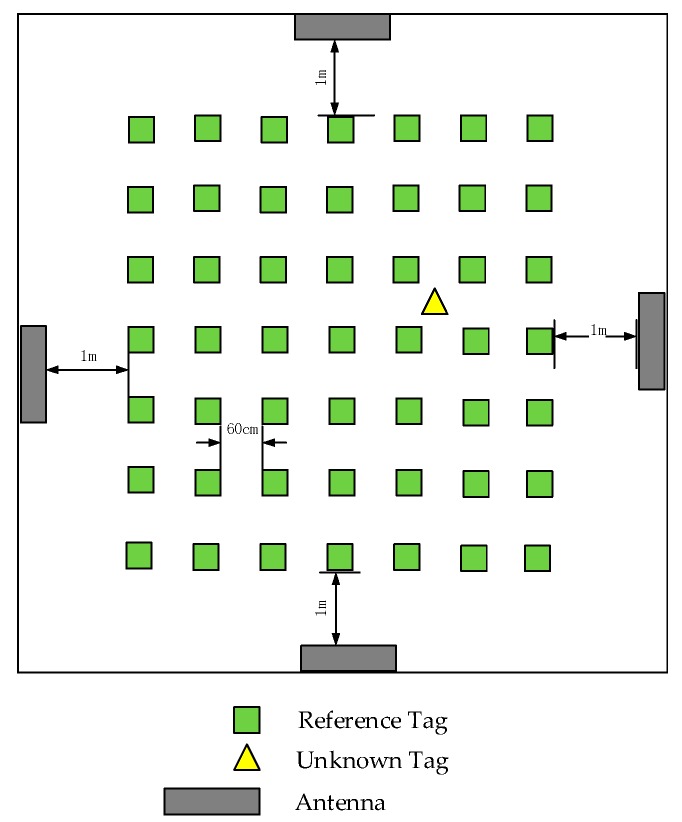
Device placement scenario.

The communication frequency between the reader and labels is between 920 MHz and 926 MHz. The feeder line is used to connect the reader and the antenna and plays the role of the data transmitter. The reader is connected to the router through WiFi. Users can operate through the computer, and the database server is installed on a personal computer. The labels are placed as shown in Figure 3 with a distance of approximately 60 cm between each adjacent reference label in each row. Figure 4 is the whole placement scenario of the device.

**Figure 5:**
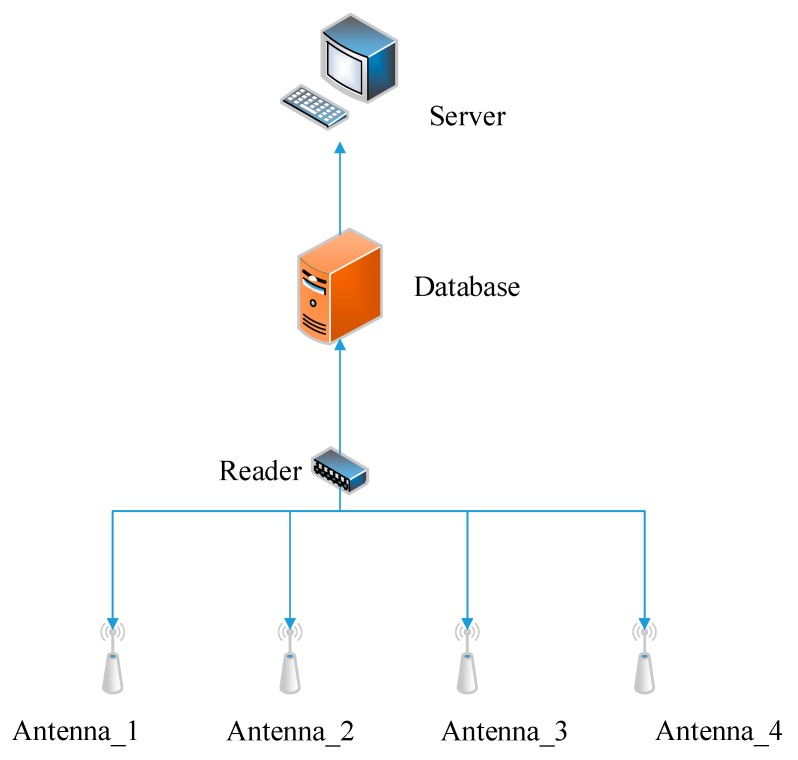
Block diagram of the RFID information acquisition module.

Figure 5 is a block diagram of the RFID information acquisition module. The monitored area is an area composed of four antennas of the same specification, and a number of the reference labels and target labels are deployed inside the area. When starting to collect RFID information, the user can control the reader through the database server, and the reader sends an instruction to the antenna to transmit the electromagnetic wave to the monitoring area. After the tag receives the electromagnetic wave, the tag transmits its own information (including RFID antenna coding, RFID tag ID number, RSSI value, phase value, etc.) fed back to the reader through the antenna to be stored in the database to complete the data acquisition. The server reads the real-time electromagnetic information (mainly the signal strength value) from the database and completes the positioning of the unknown label through several specific algorithms in this experiment.

### 5.2. Results of the Gaussian-Kalman Filter for RSSI

**Figure 6:**
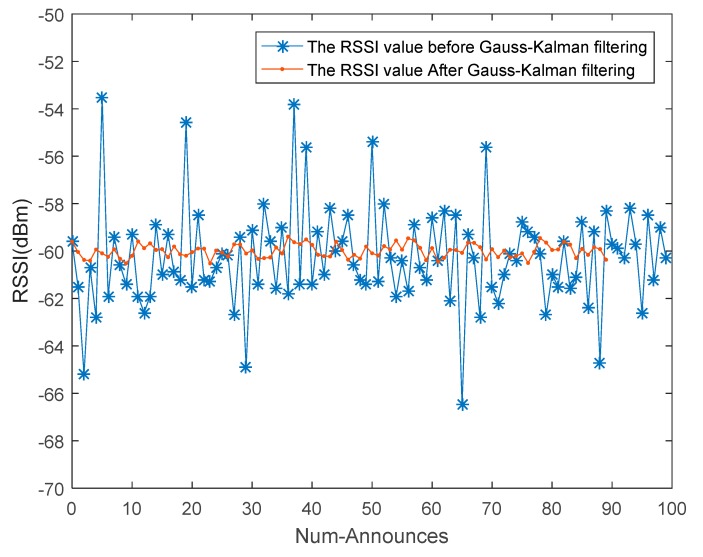
The RSSI value processed by the Gaussian-Kalman filter.

The RSSI value processing of this experiment environment is shown in Figure 5. We selected one of the antennas to read the electronic signal from the reference tag in the positioning scene to verify the effect of Gaussian-Kalman filtering. In this experiment, 100 RSSI values of the same reference tag were read, and Q = 90% was set. Gaussian-Kalman filtering data processing was as shown in Figure 6.

From Figure 6, we can see that the Gaussian fitting method is used to eliminate unstable RSSI values, and then, the Kalman filter balances the noise fluctuations of the data and achieves accurate and smooth data output.

### 5.3. Experimental Results and Analysis

There are many ways to evaluate regression algorithms. Conventional methods include mean square error, mean absolute error, root mean square error, normalized root-mean-square deviation, and so on. This paper uses a more generic root mean square error to compare experimental results. This method is also commonly used in machine learning to measure the performance and characteristics of a regression model. The root mean square error is defined as follows:

(27) $$\mathrm{RMSE}=\sqrt{\frac{1}{n}\overset{n}{\underset{i=1}{\sum }}\left[{\left(\hat{y_{i}}-y_{i}\right)}^{2}+{\left(\hat{x_{i}}-x_{i}\right)}^{2}\right]}$$

where $\hat{y_{i}}$ and $\hat{x_{i}}$ are experimental values and $y_{i}$ and $x_{i}$ are actual values. In indoor environments, *y_i_* and *x_i_* represent the actual physical position, while $\hat{y_{i}}$ and $\hat{x_{i}}$ represent the experimental position, which are obtained by using our proposed algorithm. Because the coordinates of the two dimensions are predicted separately using SVR, there are two terms in the formula. In essence, the root mean square in the two-dimensional plane is the distance between two points, and the root mean square error can visually see the distance between the predicted position and the actual position.

In this paper, 100 experiments were repeated for each of the four algorithms, and 400 experimental data were obtained. The experimental results are shown in Table 2. The original LANDMARC algorithm has the largest root-mean-square error of 35.532 cm. The SA-SVR-LANDMARC algorithm has achieved a positioning accuracy of 27.226 cm. The BPNN-LANDMARC algorithm has achieved an improvement in positioning accuracy with a root mean square error of 26.936 cm. The proposed SVR-LANDMARC algorithm has the smallest positioning error, which is 20.243 cm.

**Figure 7:**
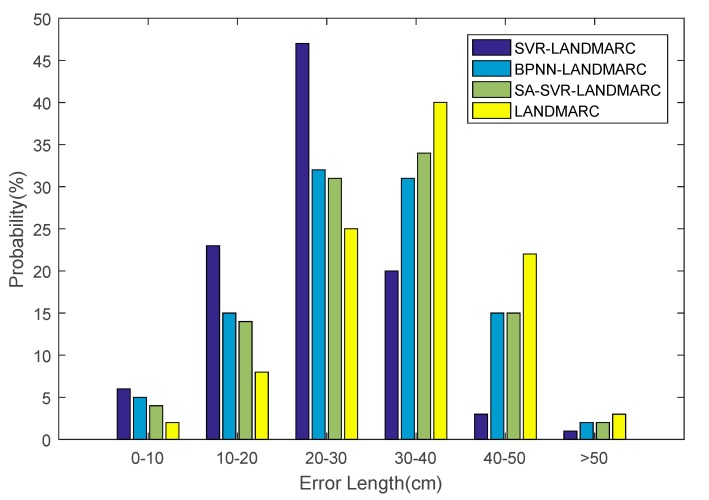
Location map of error rate distribution.

According to different classifications of the algorithm, the mapping error distance distribution map is shown in Figure 7. As can be seen from the figure, the position error results predicted by the SVR-LANDMARC indoor positioning technology are mainly distributed in 20–30 cm with a ratio of 47%; the error results predicted by the BPNN-LANDMARC indoor positioning technology are mainly distributed in 20–40 cm, The proportions of 20–30 cm and 30–40 cm were almost the same, at 32% and 31% respectively; the error length predicted by SA-SVR-LANDMARC is mainly distributed in 30–40 cm with a ratio of 34%. The SVR-based indoor positioning technology proposed in this paper has shown great improvement compared to BPNN-LANDMARC, SA-SVR-LANDMARC and the original LANDMARC. This is because the Gaussian-Kalman method can reduce the influence of some abnormal RSSI signals and make the signals smoother.

According to [29], the distance for reference tags was 1 m, but the root mean square error of positioning estimation was about 45 cm, while the root mean square error of our algorithm was about 20 cm, whereas the tag distance was 60 cm. Our positioning algorithm had nearly a 26% improvement in positioning accuracy compared to the SA-SVR-LANDMARC algorithm. In addition, in the SA-SVR-LANDMARC algorithm, four readers were used for indoor positioning, while our method used just one reader and four antennas. SA-SVR-LANDMARC used four readers to locate a 6 × 6 area. In our algorithm, just one reader was used to monitor a 3.6 × 3.6 area, which greatly reduced the use of the reader and thus reduced the system’s cost. Compared to the SA-SVR-LANDMARC technology in [29], although we used more reference tags, using less readers will also reduce the cost of deployment.

## 6. Conclusions

In this paper, a novel indoor positioning technology based on the SVR algorithm is proposed. Compared with the BPNN-LANDMARC, LANDMARC and SVR-LANDMARC algorithms, the prediction error distance is smaller. The accuracy of RFID-based indoor positioning can be improved by using the SVR algorithm and the Gaussian-Kalman filter. The Gaussian-Kalman filter has been used to solve the noise, and the SVR has been used to improve the accuracy of positioning. The proposed algorithm is very effective and suitable to be used for indoor location-based services, i.e., book searching in a library, locating patients in a hospital, finding trapped people in a burning building, finding an individual’s luggage without removing it from a vehicle or aircraft at an airport and reminding passengers about their flights and boarding gates so that they do not miss their flights or delay departures. However, the effective deployment of antennas and tags is also an important research direction in indoor positioning. How to reduce the use of tags without reducing the positioning accuracy is also a major research topic in our future work.

## Figures and Tables

**Table 1:** Different indoor positioning algorithm features.

Positioning Algorithm	Accuracy	Time Consumption	Deployment Cost	Energy Consumption	Communication Consumption
Fingerprint	High	Low	High	Low	Normal
Reference tags	Normal	High	Normal	Normal	Low
AOA	High	Normal	Normal	High	High
TOA/TDOA [10]	Low	Low	Normal	Low	High

**Table 2:** Comparisons of SVR-LANDMARC with different positioning methods.

Position Methods	Root Mean Square Error (cm)
LANDMARC [17]	35.532
SA-SVR-LANDMARC [28]	27.226
BP-LANDMARC [21]	26.936
SVR-LANDMARC	20.243
